# Lipidomic Alterations in the Mitochondria of Aged Parkin Null Mice Relevant to Autophagy

**DOI:** 10.3389/fnins.2019.00329

**Published:** 2019-04-24

**Authors:** Angel Gaudioso, Patricia Garcia-Rozas, Maria Jose Casarejos, Oscar Pastor, Jose Antonio Rodriguez-Navarro

**Affiliations:** ^1^Cellular Neurobiology Laboratory, Neurobiology Department, UCS-UCM, Hospital Universitario Ramón y Cajal, IRYCIS, Madrid, Spain; ^2^Neuropharmacology Laboratory, Neurobiology Department, Hospital Universitario Ramón y Cajal, Instituto Ramón y Cajal de Investigaciones Sanitarias (IRYCIS), Madrid, Spain; ^3^Clinical Biochemistry Department, UCA-CCM, Hospital Universitario Ramón y Cajal, IRYCIS, Madrid, Spain

**Keywords:** Parkinson, aging, mitochondrial membrane, macroautophagy, mitophagy, PARK2

## Abstract

Mitochondrial quality control is important in neurological diseases, but in genetic Parkinson’s disease caused by mutations in PINK and parkin mitochondrial degradation through autophagy is crucial. Reductions in autophagy and mitophagy are implicated in aging, age related diseases and Parkinson. The parkin null mice (PK-KO) show only a subtle phenotype, apparent with age or with stressors. We have studied the changes in the lipidomic composition of the mitochondrial membranes isolated from the brains of young and old PK-KO mice and compared them to wild type in order to determine possible implications for Parkinson’s disease pathology. We observed an increase in the levels of phosphatidylethanolamine in the young PK-KO mice that is lost in the old and correlate to changes in the phosphatidylserine decarboxylase. PK-KO old mice mitochondria showed lower phosphatidylglicerol and phosphatidylinositol levels and higher levels of some forms of hydroxylated ceramides. Regarding cardiolipins there were changes in the degree of saturation mainly with age. The lipidomic composition discriminates between the study groups using partial least square discriminant analysis. We discuss the relevance of the lipid changes for the autophagic activity, the mitophagy, the mitochondrial activity and the Parkinson’s disease pathology in absence of parkin.

## Introduction

Parkinson’s disease (PD) is a prevalent neurodegenerative disease, where genetic and environmental factors contribute to ethiopathology. Different defects are associated with PD pathogenesis, mainly, oxidative stress, mitochondrial failure and accumulation of aberrant proteins due to impairment of the proteasome or the lysosomal systems (Przedborski, 2017).

Among the PD cases due to a genetic cause, mutations in PARK-2 gene are related with early-onset autosomal recessive forms of PD (Kitada et al., 1998; Hattori et al., 2000; Dodson and Guo, 2007). PARK-2 gene encodes parkin, an E3 ubiquitin ligase of the RBR family. It was first hypothesized that the absence of degradation of specific parkin substrates by the ubiquitin–proteasome and its accumulation cause neurodegeneration (Moore et al., 2003; Klein and Schlossmacher, 2006). Characterization of the PK-KO mouse demonstrated synaptic and mitochondrial function alterations, but the death of substantia nigra neurons characteristic of PD was not evident up to the age of 24 months, meanwhile one of the most striking features of these mice was the problems to gain weight with age (Itier et al., 2003; Rodriguez-Navarro et al., 2007).

A non-neuronal function of parkin was determined when parkin was eliminated in flies (Pesah et al., 2004). These flies showed decreased body mass and defects in flight muscle mitochondria. The role of parkin in mitochondrial homeostasis has gained importance, as aging PK-KO mice also display increased oxidative stress, decreased glutathione and blunted mitochondrial respiration (Rodriguez-Navarro et al., 2007). *In vitro*, parkin translocates to de-energized mitochondria to initiate the degradation of defective mitochondria through autophagy in the process called mitophagy and has been widely implicated in the control of mitochondrial dynamics (Narendra et al., 2008, 2009, 2010).

Mitochondrial membrane lipids are essential for the mitochondrial function. The architecture, activity of respiratory proteins, transport of proteins into the mitochondria, defects in mitochondrial fission and fusion, maintenance of the double membrane structure and curvature, recruitment, amount and activity of proteins (like parkin or LC3-II, autophagosomal marker) and contribution of lipids to other organella through contacts or vesicles largely depend on the mitochondrial lipid composition (Bottinger et al., 2016; Aufschnaiter et al., 2017).

In fact, mitochondria are one of the sources for the formation of autophagosomal membranes (Hailey et al., 2010), mainly after starvation. The origin of the autophagosomal membrane is a matter of intense debate, and the lipids probably come from multiple sources (Molino et al., 2016; Morel and Codogno, 2018). Parkin could have a role in the mitochondrial lipid contribution to the autophagosomal membranes (Hailey et al., 2010; Cook et al., 2014). The exact lipid composition of those membranes is not known, but changes in the nutritional status like starvation or high fat diets affect lysosomal membrane composition and fusion of autophagosomes with Lys (Koga et al., 2010; Rodriguez-Navarro and Cuervo, 2010, 2012; Rodriguez-Navarro et al., 2012).

In addition to the intriguing decrease of age-associated weight gain, PK-KO mice fed with a high fat and cholesterol diet resisted steatohepatitis, and insulin resistance because parkin influences fatty acid uptake and metabolism (Abumrad and Moore, 2011; Kim et al., 2011; Lobasso et al., 2017). Furthermore, a siRNA screen performed in a cellular model of PD has found a link between the master regulator of lipid synthesis SREBF1 and mitophagy, underscoring the importance of lipid alterations on pathogenesis of parkin mediated PD (Ivatt et al., 2014; Ivatt and Whitworth, 2014).

Hence, the lipid composition of the mitochondria in the absence of parkin and the changes due to aging, the main risk factor for developing PD, are of great interest to understand the pathophysiology of PD and to find new targets for its treatment.

## Materials and Methods

### Animals

WT and PK-KO male mice from two age groups (young: 2 months; old: 24 months) were used (Itier et al., 2003). All procedures used in animal experiments were in accordance with legislation in Spain (RD 53/2013) and the European Directive (2010/63/EU) and approved by the Ethics Committee of the Ramón y Cajal Hospital, Madrid (ES-280790002001).

### Subcellular Fractionation Protocol

WT and PK-KO mice from two age groups (young: 2 months and old: 24 months) were starved for 6 h, sacrificed by cervical dislocation and their brains were extracted and resuspended in 0.25 M sucrose. For each fractionation protocol three mice brains per experimental group were pooled. Six independent fractionations were performed with the four experimental groups in parallel. Subcellular fractions were isolated following the method described by Marzella et al. (1982). Briefly, the isolation is achieved through a series of centrifugations including a nycodenz-density-gradient centrifugation (50, 26, 24, 20, and 15%) recovering fractions enriched in: autophagosomes at the 15–20% interface, autophagolysosomes at the 20–24% interface, and a Lys fraction was recovered in the 24–26% interface. The organelles were washed in 0.25 M sucrose, centrifuged and the pellets were resuspended in water (with protease and lipase inhibitors) and stored at −80°C (Yang et al., 2015).

### Western-Blot

Protein concentration of subcellular fractions isolate was measured by a Pierce BCA Method (Thermo Fisher Scientific #23227). 50 μg of protein were loaded onto 15% PAGE-SDS gels and electrophoresis was performed at a constant voltage of 100 V at 4°C. Transference of proteins was performed at 15 V overnight at 4°C. Antibodies used were: autophagosomal marker, anti-LC3 (MBL #PM036); lysosomal markers, anti-LAMP1 (DSHB #1D4B); anti-Cathepsin D (Abcam #ab75852); and mitochondrial markers, anti-VDAC (Cell Signaling #4661); anti-TOM20 (Cell Signaling #13929); anti-Cytochrome C (BD Pharmingen #556432). HRP-conjugated secondary antibodies were from Sigma.

### Lipidomic Analysis

For lipidomic analysis, 500 μg protein mitochondrial samples from WT and PK KO mice brains were centrifuged and resuspended in lysis buffer without detergents (TRIS 50 mM; NaF 5,3 mM; NaCl 125 mM; Na4P2O7 2.35 mM; EDTA-Na2 1 mM; Na3VO4 1 mM; Protease inhibitors). Lipid extraction was made following the Folch method (Folch et al., 1957). The lipid extract (500 μL) was split in two equal fractions. One fraction subjected to sphingolipid analysis was saponified with methanolic KOH (1M) at 37°C for 1h to remove phospholipids and the remaining fraction was used for phospholipid analysis.

Phospholipids (PC, PE, and LPC, LPE), and sphingolipids (Cer, HexCer, dhCer, SM) were analyzed using LC-MS/MS. Lipid species were separated using a Kinetex C18 column (100 Å, 1.7 μm; Phenomenex) at 55°C. A 12-min gradient from 60% solvent 1 (60% acetonitrile in water, 10 mM ammonium formate) to 100% solvent 2 (90% IPA in acetonitrile, 10 mM ammonium formate) followed by 8 min of re-equilibration was applied (flow 0.4 ml/min). Lipids detection was performed on a QTrap 4000 (AB-SCIEX) and analyzed using Analyst version 1.62. Spectra were acquired in the positive ionization mode and nitrogen was used at 500°C. Lipid extracts were dissolved in 250 μL of acetonitrile/IPA (1:1). The injection volume was 2 μL for phospholipids and 5 μL for sphingolipids. Lipids were identified by LC retention time and the pattern of MS/MS fragmentation (Chamorro et al., 2013; Busto et al., 2018). The analysis of PI, PS, PG, and CL were done by LC-MS/MS, using a Kinetex HILIC column (100 Å, 2.1 mm, 1.7 μm; Phenomenex) at 45°C using a quaternary pump gradient as described in Chamorro et al. (2013). Lipid extracts were dissolved in 100 μL of chloroform and 10 μL were injected in the system. PS species were detected by NLS at m/z 185 in positive mode. PI, PG, and CL species were detected by PIS in negative mode at m/z 241, 153 and 279.2, respectively. Internal standards used were: PE 32:2 (2500 pmol/sample); PC 28:2 (5000 pmol/sample); PS 28:0 (1000 pmol/sample); PG 34:0 (1000 pmol/sample); PI 38:0 (400 pmol/sample) LPC 17:0 (2500 pmol/sample); LPE 14:0 (1500 pmol/sample); SM 30:1 (2500 pmol/sample); Cer 37:1 (2500 pmol/sample); HexCer 33:1 (2500 pmol/sample); dhCer 35:0 (1000 pmol/sample) and CL 56:0 (200 pmol/sample). All internal standards were purchased from Avanti Polar Lipids and Matreya. Identification, quantitation and annotation of lipid species was done as described (Chamorro et al., 2013; Busto et al., 2018) following the recommendations by Liebisch et al. (2013).

### Statistical Analysis

The statistical analysis of the total lipidomic composition of mitochondria was performed using MetaboAnalyst 4.0 software (Xia et al., 2009). The original lipidomic data were subjected to missing value estimation using Probabilistic Principal Component Analysis (PPCA), normalized (mean-centered and divided by the standard deviation of each variable), analyzed using partial least square discriminant analysis (PLS-DA) and represented using a heatmap after autoscale samples. All the raw data would be available upon request. For the graph generation and the statistical analysis in the rest of the figures GraphPad Prism and Microsoft Excel were used and One way ANOVA test was performed.

## Results

### Lipidomic Composition of Mitochondrial Membranes Changes With Parkin Deletion and Age

**FIGURE 1:**
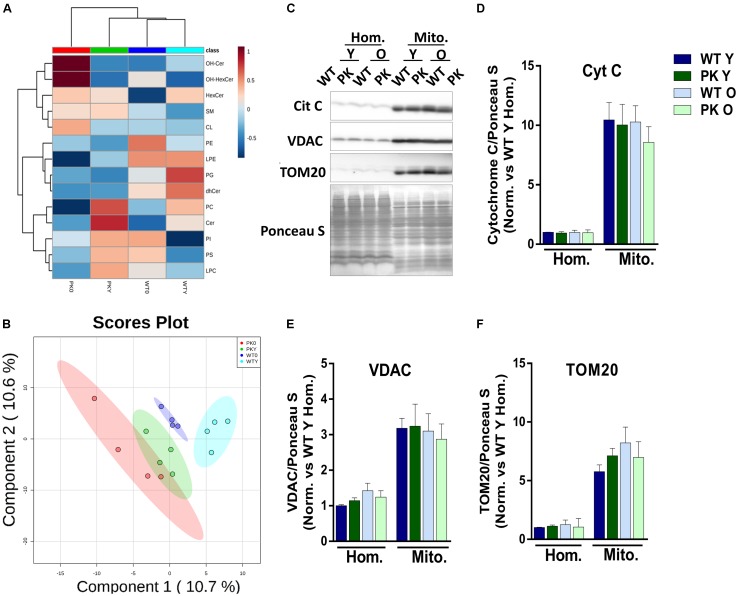
Lipidomic composition of brain’s mice mitochondria change due to aging and parkin loss. Lipidomic analysis and enrichment of mitochondrial fraction isolated from WT and PK-KO mice’s brain. **(A)** Heatmap of lipidomic classes detected and changes due to aging and parkin loss expressing the relative differences between the groups after normalization and the hierarchical clustering. **(B)** 2D representation of the Partial Least Square Discriminant Analysis of brain’s mitochondrial lipidomic composition. **(C)** Western-Blot experiments showing homogenates and mitochondria stained for Cytochrome C, VDAC, and TOM20. **(D)** Quantification of Cyt C signal intensity in homogenates and mitochondria. **(E)** Quantification of VDAC signal intensity in homogenates and mitochondria. **(F)** Quantification of TOM20 signal intensity in homogenates and mitochondria. *n* = 4–6 for western blot analysis. Graphs represent the mean ± SEM. Panels **A,B** were made using MetaboAnalyst software. Statistical analysis of western blot experiments was done by One Way ANOVA test. OH Cer, hydroxylated ceramide; OH HexCer, hydroxylated hexosylceramide; HexCer, hexosylceramide; SM, sphingomyelin; CL, cardiolipin; PE, phosphatidylethanolamine; LPE, lysophosphatidylethanolamine; PG, phosphatidylglycerol; dhCer, dihydroceramide; PC, phosphatidylcholine; Cer, ceramide; PI, phosphatiylinositol; PS, phosphatidylserine; LPC, lysphosphatidylcholine; Hom, homogenate; Mito, mitochondria; Cyt C, cytochrome C; Y, young; O, old.

The global analysis of the lipidomic composition of mitochondria is represented in Figure 1A. The hierarchical clustering indicated that the most separated mitochondria when we studied the lipid classes were the old PK-KO. They were enriched in hydroxylated forms of ceramides and presented relatively lower levels of different classes of phospholipids. As described before (Pollard et al., 2017), aging induce differences in the lipidomic composition of brain mitochondrial membranes but the changes induced by parkin elimination were more profound. When we performed partial least square discriminant analysis (PLS-DA) we were able to discriminate between the different groups due to its lipidomic composition (Figure 1B). In the Figure 1C–F the enrichment in different mitochondrial protein markers could be observed, as well as the lack of differences between WT and PK-KO regardless of the age. Even though Tom20 (Yoshii et al., 2011), cytochrome c (Gama et al., 2015) and VDAC (Geisler et al., 2010; Narendra et al., 2010) have been described as parkin E3 ubiquitin ligase substrates or key factors for parkin dependent mitophagy, they did not show accumulation in PK-KO mitochondria, not even in 24 months-old animals, meanwhile we observed multiple lipid changes. This result underlines the fact that the changes in lipids are important in the absence of parkin.

### Phosphatidylethanolamine Changes in PK-KO Mice Mitochondria Correlate With PSD

**FIGURE 2:**
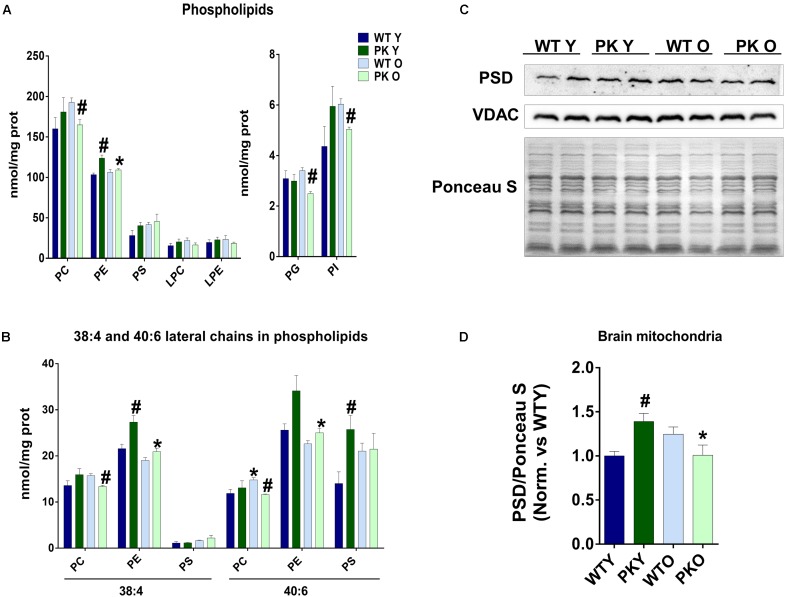
PE levels change in PK-KO mice’s mitochondria due to PSD. **(A)** Levels of phospholipids (PC, PE, PS, LPC, LPE, PG, and PI) and changes due to aging and parkin loss. **(B)** Levels of phospholipids (PC, PE, and PS) containing arachidonic (20:4) or docosahexaenoic acid (22:6) as lateral chains. **(C)** Western-blot experiment showing PSD levels in brain’s mitochondria from WT and PK-KO mice. **(D)** Quantification of PSD intensity in brain’s mitochondria. Graphs represent the mean ± SEM. One Way ANOVA statistical analysis was performed. ^∗^*p* < 0.05 due to aging. ^#^*p* < 0.05 due to parkin loss. *n* = 4 for lipidomic analysis and *n* = 6 for western-blot experiments.

The maintenance of a defined composition of mitochondrial phospholipids relies on the organelle capacity to synthesize CL, PE, PG *in situ* and on the external supply of phosphatidylcholine (PC) and PS, which are exclusively synthesized in the ER and Mitochondrial Associated Membranes (MAM) and must be imported into the mitochondria. One of the most striking changes we found in the phospholipid composition of mitochondria, was the increase in the PE levels in the PK-KO young mice (Figure 2A) due mainly to changes in the polyunsaturated forms of PE (Figure 2B). In the old PK-KO brain mitochondria, this increase was reversed and there was also less PC. Meanwhile the increase in the PS levels was not significant and the lysophospholipids did not change. PE can be synthesized using: the CDP-ethanolamine-Kennedy pathway; reacylation of lyso-PE via the Lands cycle; phosphatidyl-serine synthase-2; and decarboxylation of PS in the mitochondria by PS decarboxylase (PSD). PSD in addition to be located in the mitochondria, shows preference for the polyunsaturated forms of PS (Kevala and Kim, 2001; Bleijerveld et al., 2007). The levels of PSD enzyme correlate with the changes in PE, they were higher in young PK-KO and decreased with age (Figure 2C,D).

### PK-KO Old Mice Mitochondria Show Lower Levels of Phosphatidylglycerol and Phosphatidylinositol

**FIGURE 3:**
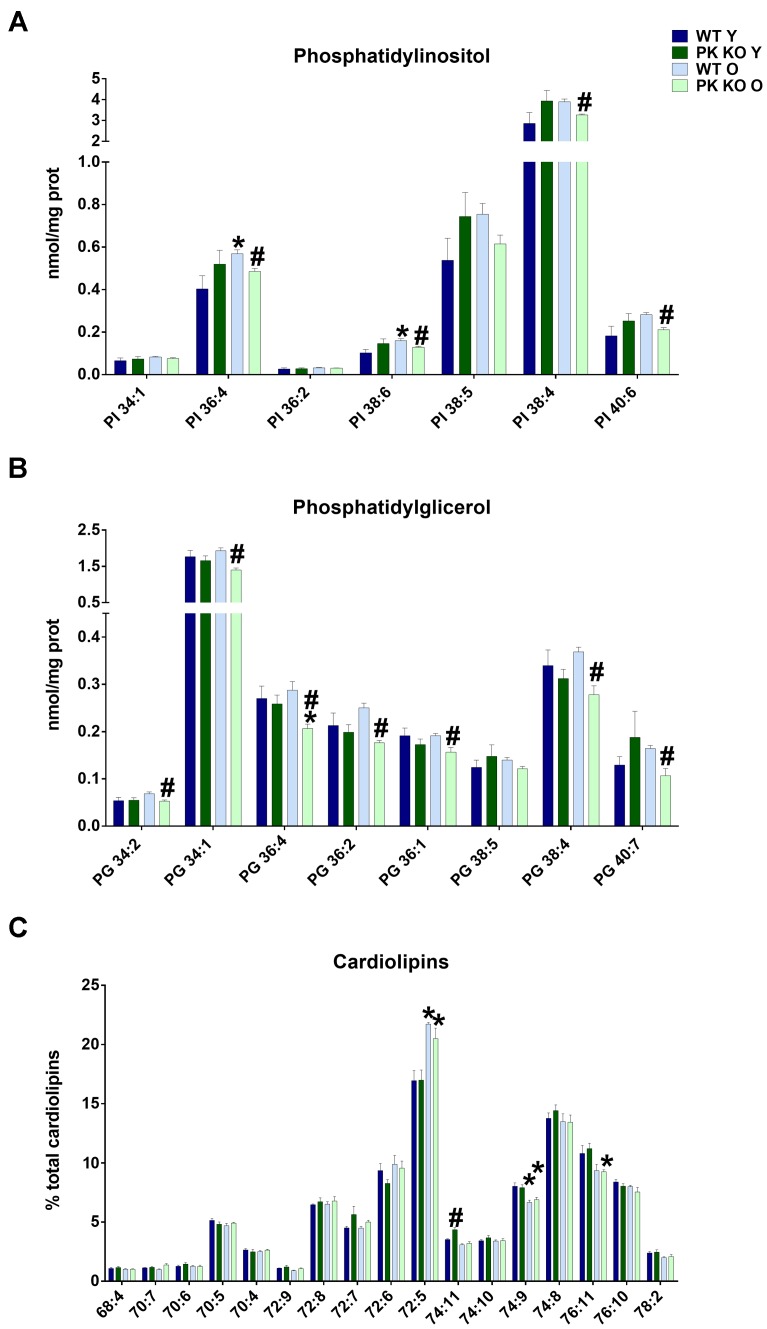
PI, PG, and Cardiolipin’s species detected in brain’s mitochondria and changes due to aging and Parkin loss. Aging decreases PG and PI levels in PK-KO mice and cardiolipin’s remodeling both in WT and PK-KO **(A)** Levels of more abundant PI species. **(B)** Levels of PG species detected. Data are expressed an nmol/mg prot for each lipid specie **(C)** Cardiolipin’s unsaturation in brain’s mitochondria. Data are expressed as percentage of each lipid specie levels versus total levels of cardiolipins detected per preparation. Graphs represent the mean ± SEM. Statistical analysis was performed by One Way ANOVA. *n* = 4 for lipidomic analysis. ^∗^*p* < 0.05 due to aging. ^#^*p* < 0.05 due to parkin loss.

We found that the levels of PI as well as PG decreased in the old PK-KO mitochondria (Figure 2A). When we studied the different PI species, we observed a significant decrease in the polyunsaturated species of PI (PI 36:4, 38:4, 38:6, and 40:6 in the Figure 3A). Phosphatidylinositol is one of the most important lipids in the process of autophagy initiation, because PI3P is involved in the recruitment of the autophagy machinery, but our technique was unable to differentiate the phosphorylated forms. However, PI3P is rarely found in the mitochondria but is enriched in the mitochondria-ER contacts and in the endosomal system (Gillooly et al., 2000).

Phosphatidylglycerol, contrarily is enriched in the mitochondrial membranes in eukaryotic cells and is the precursor of CL. In this case, all the PG species decreased (Figure 3B), including mono unsaturated (34:1 and 36:1), polyunsaturated (36:4 and 38:4) and those with linoleic acyl chain (34:2, 36:2), the main precursor of TLCL. Increasing PG levels by overexpressing an enzyme with lysophosphatidylglycerol acyltransferase or by targeted deletion of ALCAT1 stimulates mitophagy (Zhang et al., 2014; Hsu and Shi, 2016). The chronic inhibition of mitophagy in PK-KO mice could be a cause or a consequence of the lower levels of the different species of PG we found in old PK-KO mice (Figure 3B).

### Maturation of Cardiolipins Decreases With Age

In spite of the lower levels of PG, we did not find any significant differences in the absolute amount of CL due to parkin deletion. However, when we analyzed the acyl chains of CL, we observed in old WT and PK-KO mice a switch to forms with low number of unsaturations (Figure 3C). CL maintains membrane potential, architecture of the mitochondrial membrane, and the function of proteins involved in respiration. The unique structure of CL, which contains three glycerol backbones and four fatty acyl chains, makes it key for the mitochondrial function. CL acyl chains are subjected to remodeling by three enzymes: Tafazzin, monolysocardiolipin acyltransferase, and acyl-CoA:lysoCL acyltransferase. Unsaturation of CL causes deficient mitophagy in aging and genetic diseases with mutations in those enzymes, like Barth disease (Hsu and Shi, 2016). Chu et al. (2013) showed that CL remodeling influences LC3-CL direct interaction to alter mitophagy. Specifically, LC3 higher affinity was for TLCL. We did not observe changes in TLCL (72:8), but the increase in the short less unsaturated 72:5 CL forms and the decrease in the long and more unsaturated forms 74:9 and 76:11 (Figure 3C) indicated a defect in remodeling with age that could determine mitochondrial activity and autophagy. The enrichment in less unsaturated forms of CL that we found in the old brain mitochondria could represent an attempt to compensate for the loss of mitophagy due to parkin absence.

### PK-KO Old Mice Mitochondria Have Elevated Levels of Specific Forms of Hydroxylated Ceramides

**FIGURE 4:**
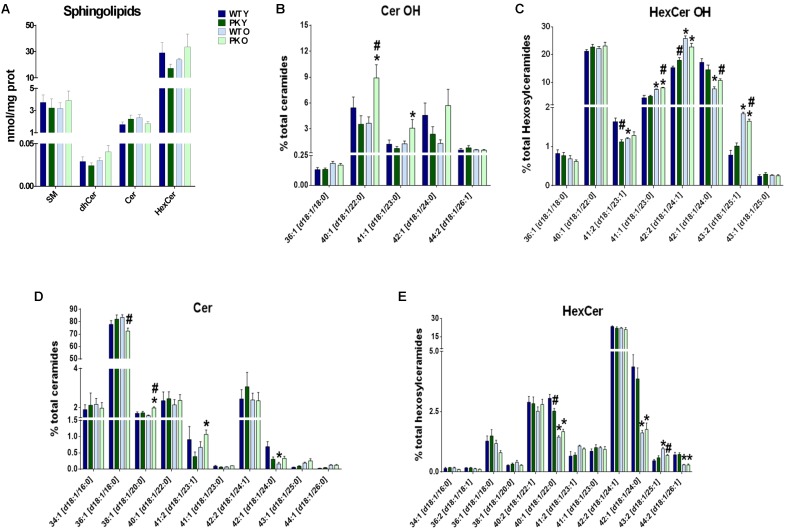
Sphingolipids content in PK-KO mice’s brain mitochondria. PK-KO mice’s mitochondria have higher levels of hydroxylated ceramides due to aging. **(A)** Levels of different classes of sphingolipids (SM, dhCer, Cer, and HexCer) and changes due to aging and parkin loss. **(B)** Percentage of Hydroxylated ceramide species. **(C)** Percentage of Hydroxylated Hexosyl Ceramide species. **(D)** Percentage of ceramide species. **(E)** Percentage of HexCer species. For panels **B–D** data are expressed as percentage of each lipid specie levels versus total levels of the lipid class analyzed. Graphs represent the mean ± SEM. Statistical analysis was performed by One Way ANOVA test. ^∗^*p* < 0.05 due to aging. ^#^*p* < 0.05 due to Parkin loss.

When we studied the changes in sphingolipids in the mitochondrial membranes, where they are minority compared to phospholipids, we did not find mayor changes due to parkin deletion or aging in the different sphingolipid classes (Figure 4A). However, some forms of hydroxy-ceramides and of hydroxy-glucosylceramides are enriched in the PK-KO old mice (Figure 4B,C), meanwhile long monounsaturated glucosylceramides decreased with age (Figure 4D,E). 2-Hydroxy fatty acid sphingolipids biosynthesis requires fatty acid 2-hydroxylase. Mutations in the fatty acid 2-hydroxylase gene, FA2H, have been associated with leukodystrophy and spastic paraparesis, underscoring the importance of OH-ceramides in the nervous system. One of hydroxylated fatty acid precursors of OH-ceramides, 2-Hydroxyoleic acid, induces autophagy (Marcilla-Etxenike et al., 2012) and the ceramide involvement in autophagy and apoptosis has been known for a long time. In fact, ceramides as well as cardiolipins have been shown to interact directly with LC3 (Sentelle et al., 2012).

### Starvation-Induced Autophagy Is Not Impaired in Young PK-KO Mice

**FIGURE 5:**
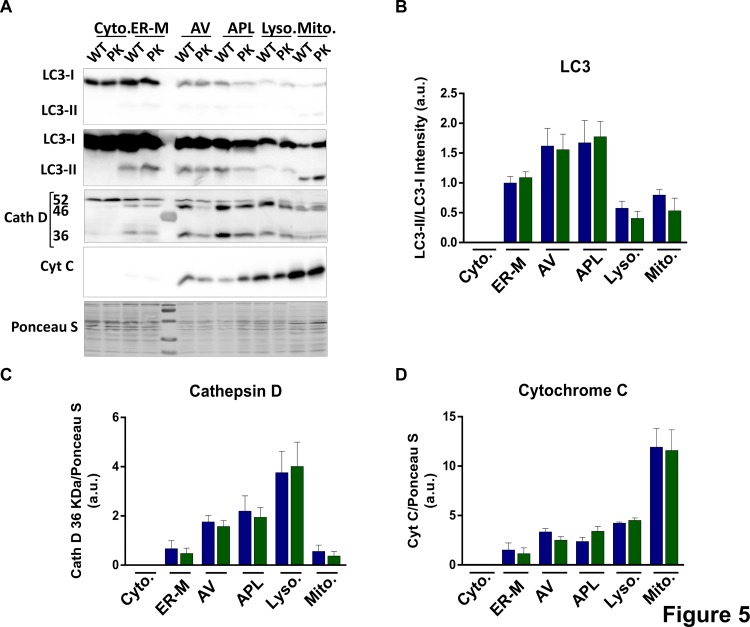
Enrichment of autophagosomes, lysosomes and mitochondria from mice’s brain. Parkin loss doesn’t impair autophagic processes in the young animals. **(A)** Representative images of Western-Blot experiments showing LC3 (LC3-I and LC3-II), (^∗^higher exposure in the bottom image), Cathepsin D (52, 46, and 36 KDa) and Cytochrome C levels in the different subcellular fractions isolated. **(B)** Quantification of LC3-II/LC3-I ratio. **(C)** Quantification of mature Cath D 36 KDa in brain’s subcellular fractions. **(D)** Quantification of Citochrome C signal intensity in brain’s subcellular fractions. Graphs represent the mean ± SEM. Statistical analysis was performed by One Way ANOVA test. *n* = 3–5 preparations of three mice each.

We have previously described that multiple lipid species including several sphingolipids, gangliosides and ceramides are elevated in autophagic vacuoles and lysosomes from brains of an Alzheimer’s disease mouse model (Yang et al., 2015). Using the same fractionation protocol, we studied if there were changes due to parkin deletion. We did not find major changes in the protein markers of autophagosomes, lysosomes or mitochondria due to parkin deletion (Figure 5A–D) in the young animals. However, in the Figure 5A we observed a shift in the electrophoretical mobility of the LC3 II band in the mitochondrial lines compared with autophagosomes.

### P62 Accumulates in the Homogenates and Autophagosomes but Not in Old PK-KO Mice Lysosomes

**FIGURE 6:**
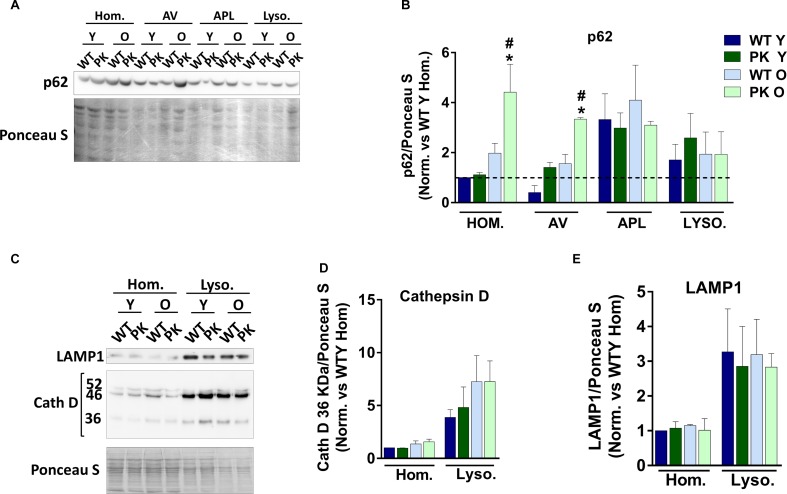
Autophagy related markers in subcelluar fractions from brain and changes due to aging and parkin loss. p62 accumulates in PK-KO old mice autophagosomes but not in autophagolysosomes or lysosomes. **(A)** Representative image of a Western-Blot experiment showing p62 levels in the different subcellular fractions isolated. **(B)** Quantification of p62 intensity in brain’s subcellular fractions. **(C)** Western-Blot experiment showing LAMP1 and Cathepsin D levels in homogenates and lysosomes. **(D)** Quantification of Cath D 36 KDa in brain’s subcellular fractions. **(E)** Quantification of LAMP1 intensity in brain’s subcellular fractions. Graphs represent the mean ± SEM. Statistical analysis was performed by One Way ANOVA test. ^∗^*p* < 0.05 due to aging. ^#^*p* < 0.05 due to Parkin loss. *n* = 3–5 preparations of three mice each.

We studied the autophagic flux in the brain *in vivo* measuring p62 in the different organelle. Sequestosome p62 is an autophagy adaptor and substrate described to be ubiquitinated by parkin for its degradation via proteasome (Song et al., 2016). In Figure 6A,B we observed an accumulation of p62 in the homogenates of PK-KO brains reflecting a lack of degradation, and in the autophagosomal fraction, indicating that it arrives to the autopaghic vacuoles after starvation. In the autophagolysosomal and in the lysosomal fractions p62 return to normal levels in the PK-KO old. In order to study lysosomal activity, we measured the levels of the processed form of the cathepsin D (Figure 6D,E). We did not find changes in the amount of active cathepsin D with age or increased in the old with PK-KO deletion in the mice brain.

## Discussion

In this work we have shown that mitochondrial lipid composition is altered with parkin deletion and with age in mouse brain. Parkin elimination in mice cause only a subtle phenotype, that worsens with age (Rodriguez-Navarro et al., 2007), stressors (Pickrell et al., 2015; Rojansky et al., 2016) or when the mice are crossed with other pathology generating mutations (Menendez et al., 2006; Rubio et al., 2009; Perucho et al., 2010). Our results indicate that major alterations in the mitochondrial lipids happen in old animals, in fact, the discriminant analysis separate the PK-KO old animals from the rest (Figure 1A). We identify specific lipidomic signatures in mitochondria with aging and parkin deletion, revealing mitochondrial brain specific lipid modifying pathways as possible targets to ameliorate age-related mitochondrial defects in absence of parkin.

### Phospatidylethanolamine and PSD Importance in Autophagy

However, in the young PK-KO mice we found an interesting increase in PE levels in the brain mitochondria similar to the one found by Valadas et al. (2014) in Drosophila. They found that mitochondria contained more PS and PE in parkin mutants, whereas the ER of parkin mutants had less PS and PE and interpret that the transfer of PS from ER to mitochondria in parkin mutants is increased. This interpretation fits well with the increase in PSD that we observed in PK-KO mice mitochondria and with the levels of the forms of PS that are PSD substrates (Figure 2). PSD is also required for adequate mitochondrial fusion and a reduction in PE impairs fusion of membranes with lipids mimicking mitochondrial composition. PE contribution from the mitochondria to the autophagosomal membranes is important for the elongation of autophagosomes during starvation (Hailey et al., 2010). PSD decrease (as the one we found with aging in PK-KO) impairs LC3 lipidation and the autophagic process, underlying how important mitochondria derived PE is for the autophagosomal membranes. Exogenously added ethanolamine or PE are able to restore autophagy in PSD knockouts, and in control cells both can activate autophagy (Chan and McQuibban, 2012). The increase in PSD we found in the young PK-KO could be responsible for the lack of defects in starvation induced autophagy, meanwhile the decrease of PSD in the old PK-KO could be responsible for the defects observed in p62 accumulation. Modulation of PSD or the levels of PE could be a therapeutic target for PD.

Decreased PE levels were described in the substantia nigra of Parkinson’s disease patients compared to control subjects (Riekkinen et al., 1975) before description of genetic forms of the disease. Meanwhile, the activity of Kennedy pathway enzymes was significantly elevated probably to compensate for low levels of PE (Ross et al., 2001). In a study of total cellular lipids from fibroblasts of parkin mutants, using MALDI-TOF and thin-layer chromatography Lobasso et al. found higher levels of gangliosides, and of LPC, PI, and PS, but clearly the total cell lipidome cannot be compared with the mitochondrial-specific changes that we present here (Lobasso et al., 2017).

### Age-Induced Changes in PK-KO Mitochondrial Lipids Relevant for Autophagy and Mitochondrial Function

The main alterations found in the brain mitochondria of PK-KO mice occurred with age, there were lower levels of PC, PG, and PI and more OH-ceramides than in the WT counterparts. Pollard et al. have described some changes in mice mitochondria due to aging in mice, focusing in the unsaturation of fatty acids (Pollard et al., 2017). Interestingly we found important differences in the unsaturation of cardiolipins with age in the brain as it happens in the heart (Lee et al., 2006).

Recently, a lysoPG acyltransferase was found to affect mitophagy. Increasing PG by overexpressing this enzyme increased autophagy and deletion of ALCAT1 also increases PG levels and activates mitophagy (Li et al., 2010; Pokorna et al., 2015). The decreased levels of PG that we found in PK-KO old mice could be responsible for a lower mitophagic activity similar to that which happened in PK-KO flies models (Cornelissen et al., 2018), although the study of the role of parkin in mitophagy *in vivo* has rendered conflicting results, depending on age and on the reporters and techniques used (Lee et al., 2017; Cornelissen et al., 2018).

Phosphatidylglycerol is mainly used in mammals mitochondria for CL synthesis (Chen et al., 2018). CL is synthesized from CDP-diacylglycerol and PG without acyl chain specificity and then is remodeled by tafazzin (an enzyme implicated in the Barth Syndrome) adding unsaturated acyl chains through successive transacylation to tetralinoleoyl CL (TLCL). CL remodeling is important for mitophagy and mitochondrial function. We find that the old mitochondria in the brain have a defect in remodeling (Figure 3C), showing low rates of unsaturation. Appropriate rates of unsaturation are required for optimal respiration in the electron transport chain and to minimize production of ROS (Hsu and Shi, 2016; Chen et al., 2018). When CL is damaged by oxidative stress, as happens with aging, it is remodeled by PLA2 followed by re-acylation by lysocardiolipin acyltransferase (ALCAT) at the MAMs or in the mitochondria by monolysoCL acyltransferase. Long and highly unsaturated fatty acyl CL is remodeled by ALCAT. ALCAT also decrease fusion-fission of mitochondrial proteins and inhibits mitophagy because CL oxidation, externalization to the outer membrane and binding to LC3 is required for proper mitophagic activity. LC3 binding to CL depends on the CL acyl chains, and showed greater affinity for TLCL (Chu et al., 2013). It has been described that LC3 interacts with different forms of cardiolipin *in vitro* (Sentelle et al., 2012; Chu et al., 2013; Antón et al., 2016). We observed a change in the LC3 mobility in the brain mitochondria on our samples similar to the one showed before in a mice model of Alzheimer’s disease (Yang et al., 2015). This change in mobility could be due to alterations in the processing of LC3, to a conjugation with a different lipid or to posttranslational modifications. *In vitro*, LC3 could be conjugated to PS and ceramides in addition to PE, depending on pH and interaction with anionic phospholipids (Nakatogawa et al., 2008; Oh-oka et al., 2008). It is tempting to speculate that due to the externalization of cardiolipin and the higher pH in the vicinity of mitochondria, the band shift that we observed *in vivo* could represent a conjugation to a different lipid, namely PS or ceramides. Cardiolipin exposure to the outer membrane not only affects mitophagy, it also modulates α-synuclein aggregation and affects the electron transport (Ryan et al., 2018).

Regarding sphingolipids in the mitochondrial membranes, some forms of hydroxy-ceramides and of hydroxy-glucosylceramides are enriched in PK-KO old mice. Genetic defects in the biosynthesis and degradation of hydroxy fatty acids cause neurodegenerative diseases. 2-hydroxy sphingolipids biosynthesis requires fatty acid 2-hydroxylase. Mutations in the fatty acid 2-hydroxylase are associated with hereditary spastic paraparesis, and mutations in lysosomal acid ceramidase (that removes the headgroup from 2-hydroxy sphingolipids in the Lys), cause a lysosomal disorder, Farber’s disease (Hama, 2010). Both diseases present autophagic problems and neurodegeneration. In the absence of parkin there is more oxidative stress (Rodriguez-Navarro et al., 2007) and OH radicals, which could drive the higher formation of these uncommon lipid species in PK-KO old mice.

Relevance of the lipid changes described here on mitophagy *in vivo* is difficult to assess, but with the new mice mitophagy reporter models it could be studied in the future, in spite of the caveats of the model (McWilliams and Ganley, 2019). We have isolated the organelles involved in the process, with a protocol that allow the enrichment in the different subcellular fractions, and we did not find mayor differences for the main organelle markers due to parkin elimination in the young mice (Figure 5). It will be interesting to determine lipidomic changes in the different organelles to study the traffic and activity of the authophagic process in conditions of activation of autophagy or in disease conditions, even though the enrichment of vesicles in the brain present the additional problem of separate the synaptic vesicles.

Contrarily to other markers, with the p62 adaptor we observed an accumulation in the homogenates that is reverted through the autophagic compartments upon starvation (Figure 6). As p62 is a substrate ubiquitinated by parkin for its degradation via proteasome (Song et al., 2016), it reinforces the idea that in the absence of parkin, starvation-induced autophagy could be upregulated in the young mice, as happened in embryonic neuronal and glial cell cultures (Casarejos et al., 2009), but not in the old ones. The normalization of p62 in the autophagolysosomes and lysosomes could mean that the fusion between AVs and lysosomes is impaired in the old PK-KO mice. Some of the changes that we observe in the old PK-KO mitochondria (less PG and PI and more OH-ceramides) could be present also in the membranes of lysosomes or autophagosomes altering its fusion ability (Koga et al., 2010; Rodriguez-Navarro and Cuervo, 2012).

The lipidomic characterization of the mitochondria, ER, autophagosomes and lysosomes in different conditions would allow us to understand better the lipid trafficking process happening during mitophagy and starvation-induced autophagy in health and disease.

The possible interventions in order to avoid the age related pathology in PK-KO identified in this study include approaches to activate PSD, or to avoid the observed decrease in PG and PI, as well as modifiers of cardiolipin remodeling. In conclusion, we identify here multiple lipid alterations in the absence of parkin that could help us understand the molecular basis of the disease and find new therapeutic targets to stimulate the autophagic process and revert the aggregation of toxic proteins in Parkinson’s disease pathology.

## Ethics Statement

All procedures associated with animal experiments were in accordance with Spanish legislation (RD 53/2013) and the European Union Council Directive (2010/63/EU) and approved by the Ethics Committee of the Hospital Ramón y Cajal, Madrid (Animal Facilities ES280790002001).

## Author Contributions

JR-N conceived and designed the experiments. AG, PG-R, and JR-N performed the experiments. JR-N, AG, OP, and MC analyzed the data. OP and MC contributed reagents, materials, analysis tools. JR-N and AG wrote the manuscript. JR-N, AG, OP, and MC revised and edited.

## Conflict of Interest Statement

The authors declare that the research was conducted in the absence of any commercial or financial relationships that could be construed as a potential conflict of interest.

## Abbreviations

APL: autophagolysosomal vesicles
AV: autophagosomal vesicles
Cer: ceramide
CL: cardiolipin
Cyt: cytosol
dhCer: dihydroceramide
ER: endoplasmic reticulum
HexCer: hexosylceramide
LPC: lysophosphatidyl choline
LPE: lysophosphatidyl ethanolamine
Lys: lysosome
MAM: mitochondrial associated membrane
Mito: mitochondria
PC: phosphatidylcholine
PD: parkinson’s disease
PE: phosphatidylethanolamine
PG: phosphatidylglycerol
PI: phosphatidylinositol
PK-KO: parkin knock-out
PS: phosphatidylserine
PSD: phosphatidylserine decarboxylase
SM: sphingomyelin
TLCL: tetralinoleoyl cardiolipin.
